# The association of frailty on cardiac rehabilitation goal achievement

**DOI:** 10.3389/fcvm.2024.1441336

**Published:** 2024-08-13

**Authors:** Evan MacEachern, Jack Quach, Nicholas Giacomantonio, Olga Theou, Troy Hillier, Wanda Firth, Dustin Scott Kehler

**Affiliations:** ^1^ School of Physiotherapy, Dalhousie University, Halifax, NS, Canada; ^2^ Faculty of Health, Dalhousie University, Halifax, NS, Canada; ^3^ Department of Cardiology, Dalhousie University, Halifax, NS, Canada; ^4^ Division of Geriatric Medicine, Dalhousie University, Halifax, NS, Canada; ^5^ Department of Surgery, Dalhousie University, Halifax, NS, Canada; ^6^ Hearts & Health in Motion, Nova Scotia Health, Halifax, NS, Canada

**Keywords:** cardiac rehabilitation, frailty, frailty index, cardiovascular, goal-setting

## Abstract

**Introduction:**

Frailty is common among patients entering cardiac rehabilitation (CR). Frailty is associated with poor health outcomes; however, it is unclear if frailty influences achieving goals in CR.

**Methods:**

We report a secondary analysis of participants who were referred to an exercise and education-based CR program from 2005 to 2015. Frailty was measured by a 25-item accumulation of deficits frailty index (FI) ranging from 0 to 1; higher scores indicate higher frailty. Participants were categorized by admission frailty levels (FI scores: < 0.20, 0.20–0.29, 0.30–0.39, > 0.40). CR goals were determined with shared decision-making between CR staff and the patients. We conducted logistic regression analyses to examine the odds of goal attainment by CR completion, adjusting for age, sex, education, marital status, and referring diagnosis. Analyses were performed using baseline frailty as a categorical and continuous outcome, and frailty change as a continuous outcome in separate models.

**Results:**

Of 759 eligible participants (age: 59.5 ± 9.8, 24% female), 607 (80%) participants achieved a CR goal at graduation. CR goals were categorized into similar themes: control or lose weight (*n* = 381, 50%), improve physical activity behaviour and fitness (*n* = 228, 30%), and improve cardiovascular profile (*n* = 150, 20%). Compared to the most severe frailty group (FI >0.40), lower levels of frailty at baseline were associated with achieving a goal at CR completion [FI < 0.20: OR = 4.733 (95% CI: 2.197, 10.194), *p* < .001; FI 0.20–0.29: OR = 2.116 (1.269–3.528), *p* = .004]. Every 1% increase in the FI was associated with a 3.5% reduction in the odds of achieving a CR goal [OR = 0.965 (0.95, 0.979), *p* < .001]. Participants who reduced their frailty by a minimally clinically important difference of at least 0.03 (*n* = 209, 27.5%) were twice as likely to achieve their CR goal [OR = 2.111 (1.262, 3.532), *p* = .004] than participants who increased their frailty by at least 0.03 (*n* = 82, 10.8%). Every 1% improvement in the FI from baseline to follow up was associated with a 2.7% increase in the likelihood of CR goal achievement [OR = 1.027 (1.005, 1.048), *p *= .014].

**Conclusion:**

Lower admission frailty was associated with a greater likelihood of achieving CR goals. Frailty improvements were associated with CR goal achievement, highlighting the influence of frailty on goal attainment.

## Graphical Abstract

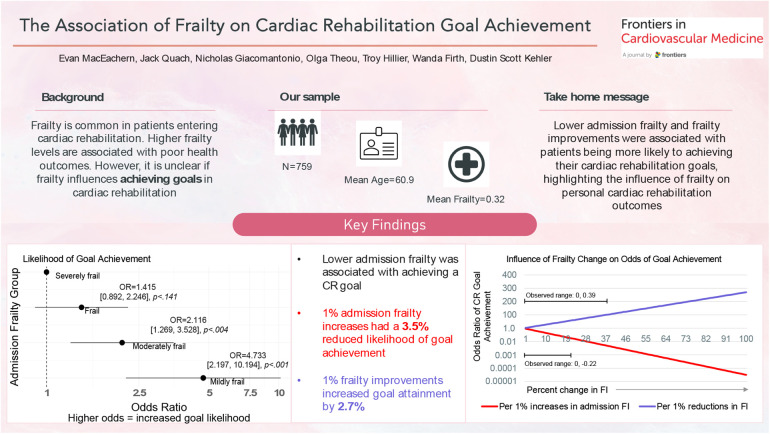

## 1 Introduction

Cardiovascular diseases (CVDs) are a major global concern, where there are high rates of morbidity and mortality (1). The standard of care to combat the progression of CVD is cardiac rehabilitation (CR) (2). The cornerstones of CR are education and exercise training, goal setting, medication recommendations, nutritional counselling, psychosocial support, and cardiovascular risk factor reduction (2, 3). These core components are delivered by a multidisciplinary team who guide program assessment and supervision, exercise prescription, and shared goal-setting between CR staff and the patient (2–4). The purpose of goal-setting in CR is to improve the participant's health behaviours, interaction with education material, and improve their health and quality of life (5). Establishing realistic and relevant goals can promote autonomy and self-efficacy in managing one's health to prolong independence and well-being (3, 6, 7).

Goal-setting can be a challenge for older people living with CVD who have multiple, interacting health deficits that require treatment through multiple healthcare resources and health services (8). Frailty is a way to capture the health of people as they age (9, 10), and is characterized by the pace at which an individual will accumulate health problems over time (10, 11). People with CVD have high levels of frailty (12), which is also observed in CR (13). Enrollment in CR can have significant benefits for managing CVD, and improving frailty levels (13–16). Here, we examine the association of frailty with achieving goals created through shared decision making between CR staff and patients. We hypothesize that higher levels of frailty will be associated with a reduced probability of achieving CR goals. We also predict that improving frailty from admission to CR completion would be associated with CR goal attainment. Determining if frailty impacts CR participants' ability to achieve CR goals will strengthen our understanding of success in CR, and help to identify those requiring greater support.

## 2 Methods

### 2.1 Study design

This is a secondary analysis of data collected as a part of routine care at the Nova Scotia Health—Hearts and Health in Motion CR program in Halifax, Nova Scotia.

### 2.2 Sample

The study sample was drawn from 4,004 former male and female CR participants aged 18 years or older who enrolled in the Nova Scotia Health CR program from May 2005 to April 2015 (13, 16). All participants included in this sample were referred to CR by a specialist (e.g., cardiologist, cardiac surgeon via automatic discharge of inpatients) or primary care provider who had a diagnosis of coronary artery disease (CAD), myocardial infarction (MI), percutaneous coronary intervention (PCI), coronary artery bypass or valve surgery (CABG), heart failure (HF), or a combination of “other” diagnoses with low rates of referral. To be included in this analysis, participants had to have set a goal based on shared decision-making with CR staff, complete the 12-week CR program, an indication if the goal was met or not, and had no missing information on education, marital status, referring diagnosis, employment status, age, sex, or baseline frailty score.

### 2.3 Cardiac rehabilitation program

The CR program was a 12-week, group-based, exercise and education intervention delivered at a single hospital-affiliated center in an urban community. The CR program sought to improve cardiovascular health through lifestyle modifications by increasing physical activity levels, improving diet, and medication recommendations if required. Behavior modification strategies were also implemented, including goal setting. The CR program was led by a multidisciplinary team, consisting of a nurse, dietitian, physiotherapist, medical director, and program lead, upholding a participant-to-staff ratio of 7:1 (Supplementary Table S1). Volume of rehabilitation consisted of exercise sessions up to twice weekly and education sessions once weekly. Duration of the exercise sessions was 60 min, which included a warm-up and cool-down, with 40 min allocated to aerobic-based exercise (i.e., cycle or arm ergometer) and 10 min allocated for resistance exercise training (i.e., dumbbell, resistance band, and body weight exercises). Participants also received an individualized, home-based aerobic and resistance training program as a supplement prescribed by a physiotherapist. Participants were advised to exercise at moderate intensity and managed workload by self-monitoring heart rate and their rate of perceived exertion (Borg RPE of 11–13 out of 20).

### 2.4 Frailty index

Frailty was assessed by a 25-item deficit accumulation frailty index (FI) developed in accordance with previously published guidelines (17). FI variables were collected by CR staff (i.e., nurse, dietician, or physiotherapist) at baseline (week one) of the CR program and upon completion of the program at 12 weeks from baseline. Variables included cardiovascular biomarkers (i.e., triglycerides, low-density lipoprotein cholesterol, and high-density lipoprotein cholesterol, total cholesterol, fasting blood glucose, systolic and diastolic blood pressure, resting pulse rate, pulse pressure, and mean arterial pressure), symptoms (i.e., New-York Heart Association functional class), quality of life according to the SF-36 questionnaire (i.e., physical, mental, and general health domains), cardiovascular fitness (i.e., peak metabolic equivalents on an exercise stress test), body composition according to body mass index, waist circumference, and bioelectrical impedance (i.e., percent fat mass and percent lean mass), and diet as determined by the Food Frequency Questionnaire (Supplementary Table S2). The individual health deficits and cut points to define frailty are described elsewhere (13). Each FI variable provided a score ranging from 0 (health deficit absent) to 1 (health deficit present) using an incremental grading scale for variables with multiple outcomes. Frailty was based on the ratio of health deficits present in the individual. For example, participants who had 10/25 items were given a frailty score of 0.4—higher ratios indicate higher levels of frailty. Continuous frailty scores and scores using clinically meaningful cut-points were used to categorize frailty (FI scores of <0.20, 0.20–0.29, 0.30–0.39, >0.40). Additional covariates included in this analysis were sex (i.e., male, female), level of education (i.e., no high school, high school education, community college, trade, some university, bachelor's degree, graduate education or higher), referring diagnosis (i.e., CAD, MI, PCI, bypass or valve surgery, HF, or “other”), employment status (i.e., disability, sick leave, unemployed, part-time, full-time, or retired) and marital status (i.e., divorced, widowed, single, married).

### 2.5 Outcome

The primary outcome was CR goal attainment upon completion of the 12-week centre-based CR program. Participants established their CR goals using the SMART goals framework (18) and through shared-decision making with the physiotherapist, dietitian, and nurse via qualitative interviewing at CR admission. All participants determined a primary CR goal between week one (baseline) and week three of the program. Result of goal achievement was assessed upon completion of the CR program at 12-weeks during the discharge assessment and data collection with CR staff (i.e., team physiotherapist, dietician, or nurse). Participants’ goal results were dichotomized into a binary distinction (yes or no) of whether the goal was met or not met at 12-weeks. Determination of goal achievement was decided by CR staff (i.e., team physiotherapist, dietician, or nurse) who were assigned to the participant during their CR program. We defined participant's CR completion as attending at least 65% of CR sessions and completing data collection at discharge (13).

### 2.6 Statistical analysis

All statistical analyses were performed using IBM SPSS Statistics 29 Software. Descriptive statistics (Table 1) on frequencies of demographic predictor variables were calculated with the Chi-square test. Frequency of CR goal achievement based on type of CR goal (Table 1, Supplementary Table S3) was calculated using a Chi-square test. Binary logistic regression analysis examined the outcome of achieving participants' goal. Binary logistic regression analysis used a fully-adjusted model with predictor variables, which age, sex, education, marital status, employment status, referring diagnosis, and frailty. Separate fully-adjusted models were completed: (1) admission frailty as a continuous score; (2) admission frailty based on clinically meaningful categories in 0.1 increments (3) frailty change as a continuous variable; (4) and frailty change based on a minimally clinically important difference [≥0.03 frailty increase, no change (±0.03), ≥0.03 frailty reduction] (19, 20). As BMI is linked to weight loss, we excluded BMI from our FI in a sensitivity analysis (Supplementary Table S8).

**Table 1:** Demographic information on sample.

Demographics	Study sample	Goal outcome		Admission frailty levels			
		Achieved	Not achieved	<0.20	0.20–0.29	0.30–0.39	>0.40
*Total (N, %)*	*759 (100.0%)*	*607 (79.9%)*	*152 (20.1%)*	*129 (17.0%)*	*207 (27.3%)*	*219 (28.9%)*	*204 (26.9%)*
Sex							
Male	580 (76.4%)	479 (82.6%)	101 (17.4%)	113 (87.6%)	165 (79.7%)	169 (77.2%)	133 (65.2%)
Female	179 (23.6%)	128 (71.5%)	51 (28.5%)	16 (12.4%)	42 (20.3%)	50 (22.8%)	71 (34.8%)
Mean age^a^ (N, SD)	60.96 (10.84)	61.4 (10.76)	59.2 (10.97)	60.9 (10.21)	60.8 (11.81)	61.0 (10.82)	61.1 (10.25)
Frailty score							
<0.2	129 (17.0%)	120 (93.0%)	9 (6.9%)	–	–	–	–
0.20–0.29	207 (27.3%)	174 (84.0%)	33 (15.9%)	–	–	–	–
0.30–0.39	219 (28.9%)	170 (77.6%)	49 (22.3%)	–	–	–	–
>0.40	204 (26.9%)	143 (70.1%)	61 (29.9%)	–	–	–	–
Mean baseline frailty score (N, SD)							
*Total*	*0.32 (0.12)*	*0.31 (0.12)*	*0.37 (0.12)* *	0.14 (0.04)	0.25 (0.02)	0.34 (0.03)	0.48 (0.06)
<0.2	0.14 (0.04)	0.14 (0.04)	0.13 (0.03)	–	–	–	–
0.20–0.29	0.25 (0.02)	0.25 (0.02)	0.25 (0.03)	–	–	–	–
0.30–0.39	0.34 (0.03)	0.35 (0.02)	0.34 (0.03)	–	–	–	–
>0.40	0.48 (0.06)	0.47 (0.06)	0.50 (0.07)	–	–	–	–
Cardiac rehabilitation goal							
Control or lose weight	381 (50.2%)	251 (65.8%)	130 (34.2%)	35 (27.1%)	80 (38.6%)	124 (56.6%)	142 (69.6%)
Physical activity behaviour and fitness	228 (30.0%)	216 (94.7%)	12 (5.3%)	60 (46.5%)	86 (41.5%)	49 (22.4%)	33 (16.2%)
Improve CV profile	150 (19.8%)	140 (93.3%)	10 (6.7%)	34 (26.4%)	41 (19.8%)	46 (21.0%)	29 (14.2%)
Education							
No high school	151 (19.9%)	117 (77.5%)	34 (22.5%)	18 (14.0%)	36 (17.4%)	43 (19.6%)	54 (26.5%)
High school	167 (22.0%)	126 (75.4%)	41 (24.6%)	27 (20.9%)	44 (21.3%)	58 (26.5%)	38 (18.6%)
Community college/trade	237 (31.2%)	186 (78.5%)	51 (21.5%)	38 (29.5%)	68 (32.9%)	66 (30.1%)	65 (31.9%)
Some university	130 (17.1%)	110 (84.6%)	20 (15.4%)	27 (20.9%)	35 (16.9%)	38 (17.4%)	30 (14.7%)
Bachelor's degree	74 (9.7%)	68 (91.9%)	6 (8.1%)	19 (14.7%)	24 (11.6%)	14 (6.4%)	17 (8.3%)
Postgraduate	0 (0.0%)	0 (0.0%)	0 (0.0%)	0 (0.0%)	0 (0.0%)	0 (0.0%)	0 (0.0%)
Diagnosis^a^							
CAD	232 (30.6%)	191 (82.3%)	41 (17.7%)	44 (34.1%)	54 (26.1%)	65 (29.7%)	69 (33.8%)
PCI	59 (7.8%)	40 (67.8%)	19 (32.2%)	8 (6.2%)	19 (9.2%)	17 (7.8%)	15 (7.4%)
Surgery	146 (19.2%)	116 (79.5%)	30 (20.5%)	24 (18.6%)	50 (24.2%)	38 (17.4%)	34 (16.7%)
HF	57 (7.5%)	42 (73.7%)	15 (26.3%)	4 (3.1%)	14 (6.8%)	15 (6.8%)	24 (11.8%)
MI	252 (33.2%)	208 (82.5%)	55 (17.5%)	47 (36.4%)	65 (31.4%)	83 (37.9%)	57 (27.9%)
Other	13 (1.7%)	10 (76.9%)	3 (23.1%)	2 (1.6%)	5 (2.4%)	1 (0.5%)	5 (2.5%)
Employment status							
Disability	47 (6.2%)	31 (66.0%)	16 (34.0%)	4 (3.1%)	6 (2.9%)	16 (7.3%)	21 (10.3%)
Sick leave	17 (2.2%)	14 (82.4%)	3 (17.6%)	1 (0.8%)	3 (1.4%)	5 (2.3%)	8 (3.9%)
Unemployed	34 (4.5%)	30 (88.2%)	4 (11.8%)	4 (3.1%)	11 (5.3%)	9 (4.1%)	10 (4.9%)
Part-time	155 (20.4%)	129 (83.2%)	26 (16.8%)	33 (25.6%)	47 (22.7%)	39 (17.8%)	36 (17.6%)
Full-time	333 (43.9%)	267 (80.2%)	66 (19.8%)	61 (47.3%)	94 (45.4%)	96 (43.8%)	82 (40.2%)
Retired	173 (22.8%)	139 (78.6%)	37 (21.4%)	26 (20.2%)	46 (22.2%)	54 (24.7%)	47 (23.0%)
Marital status							
Divorced	56 (7.4%)	39 (69.6%)	17 (30.4%)	7 (5.4%)	11 (5.3%)	16 (7.3%)	22 (10.8%)
Widowed	52 (6.9%)	46 (88.5%)	6 (11.5%)	8 (6.2%)	17 (8.2%)	14 (6.4%)	13 (6.4%)
Single	49 (6.5%)	34 (69.4%)	15 (30.6%)	6 (4.7%)	15 (7.2%)	14 (6.4%)	14 (6.9%)
Married	602 (79.3%)	488 (81.1%)	114 (18.9%)	108 (83.7%)	164 (79.2%)	175 (79.9%)	155 (76.0%)

^a^ CAD, coronary artery disease; CV, cardiovascular; HF, heart failure; MI, myocardial infarction; N, number; PCI, percutaneous coronary intervention; QOL, quality of life; SD, standard deviation.

* Alpha set at .05.

## 3 Results

### 3.1 Participant characteristics

**Figure 1:**
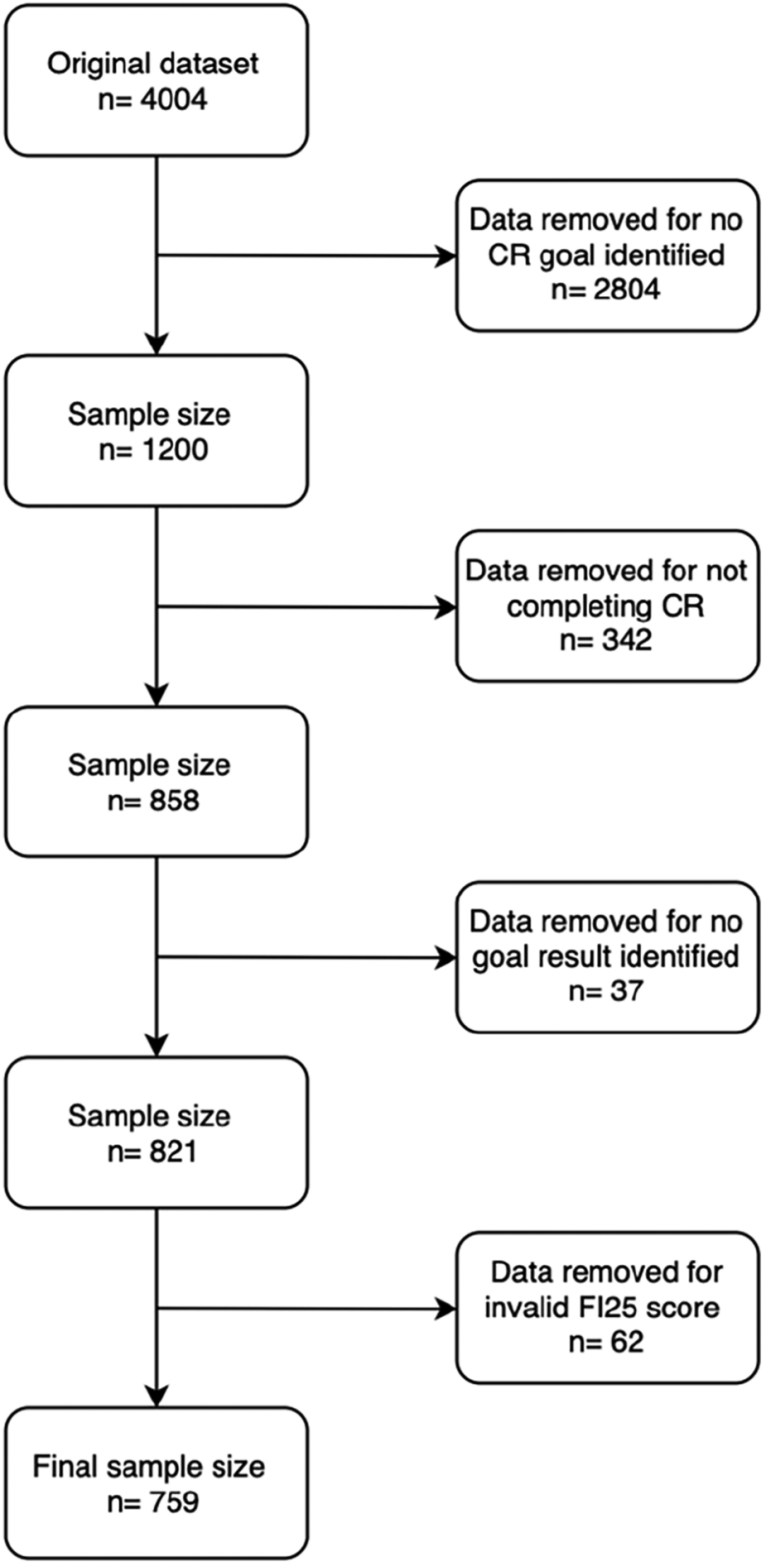
Flowchart of study inclusion.

Of the original 4,004 participants who enrolled in CR from 2005–2015, 1,200/4,004 (29.9%) met our inclusion criteria. Of these participants, 342/1,200 (28.5%) were removed for not completing the CR program. Missing information regarding the result of the CR goal removed an additional 37/858 (4.3%) participants. Lastly, 62/821 (7.5%) were removed for having a missing FI score, resulting in 759 participants included in our study (Figure 1). Additional details on the demographic information of individuals who did not complete the CR program can be found in Supplementary Table S4.

Our sample of 759 CR participants consisted of 76.4% male participants with a mean age of 60.9 (SD 10.8). Common demographic features of our sample included diagnoses of MI and CAD, education backgrounds in community college or trades; the majority of participants had full-time employment, and were married (Table 1). A total of 607 (79.9%) participants achieved their personal goal in CR. Common goals were related to controlling or losing weight, physical activity behaviour and fitness, and improving cardiovascular profile (Table 1). Participant frailty levels were high at CR admission [mean: 0.32 (SD 0.12)], with a total of 219 (28.9%) and 204 (26.9%) participants who were moderately frail (FI 0.30–0.39) and severely frail (FI >0.4) respectively (Table 1). Those who did not achieve a CR goal had greater frailty levels [FI = 0.37 (0.12), *p < *.001] compared to the mean, while goal achievers were similar to the sample mean [FI = 0.31 (0.12), *p *= .126]. Furthermore, participants who were frailer at CR admission (i.e., FI = 0.30–0.39, >0.40) had a moderate to large improvements (i.e., FI reductions of 0.09–0.15 and >0.15, respectively) over the course of CR, as compared to lower baseline FI's. The lower admission FI group (i.e., FI < 0.20) was less likely to change their FI scores at CR completion (Supplementary Table S5).

### 3.2 Characteristics associated with CR goal achievement

Admission variables associated with CR goal achievement were lower frailty at CR admission; being male; being older in age; having a bachelor's degree compared to no high school education; and a referral diagnosis of PCI rather than CAD (Table 2). Compared to participants who were severely frail, (i.e., FI >0.40), those with lower baseline FIs of <0.20 [OR = 4.733 (95% CI: 2.197, 10.194), *p < *.001] or 0.20–0.29 [OR = 2.116 (95% CI: 1.269, 3.528), *p *= .004] had a significantly greater likelihood of achieving a CR goal upon program completion. When analyzing baseline FI as a continuous variable, we observed a 3.5% reduced likelihood of achieving a CR goal for every 1% increase in admission FI score [OR = 0.965 (95% CI: 0.950, 0.979); Table 2]. Those who reduced their FI score by a minimally clinically important difference of at least 0.03 from admission to completion of CR were twice as likely to achieve their CR goal [OR = 2.111 (1.262, 3.532), *p *= .004]. Every 0.01-unit reduction in the FI was associated with a 2.7% increased likelihood of CR goal achievement upon program completion [OR = 1.027 (1.005, 1.048), *p *= .014]. Finally, sex and age-based comparisons revealed that female participants were significantly less likely to achieve a CR goal compared to males [OR = 0.616 (95% CI: 0.399–0.951), *p *= 0.029], and the likelihood of achieving a CR goal increased by 3% with every year aged [OR = 1.03 (95% CI: 1.012, 1.063), *p *= 0.004; Table 2]. Our sensitivity analysis determined the exclusion of BMI did not affect the outcome of the frailty-goal achievement relationship.

**Table 2:** Odds of achieving a CR goal according to admission factors.

Variable	Adjusted odds ratio (OR, 95% CI)	*p*-value
Sex		
Male	1.00 (ref)	
Female	0.616 (0.399, 0.951)	.029*
Age (per 1-year increase)	1.03 (1.012, 1.063)	.004*
Admission frailty level		
Per 0.01 increase	0.965 (0.950, 0.979)	<.001*
<0.2	4.733 (2.197, 10.194)	<.001*
0.20–0.29	2.116 (1.269, 3.538)	.004*
0.30–0.39	1.415 (0.892, 2.246)	.141
>0.40	1.00 (ref)	
Frailty improvement group		
>0.09–0.03 frailty increase	1.00 (ref)	(ref)
±0.03 no frailty change	1.408 (0.772, 2.569)	.264
≥0.03 frailty reduction	2.111 (1.262, 3.532)	.004*
Frailty improvement per 0.01-unit change	1.027 (1.005, 1.048)	.014*
Education		
No high school	1.00 (ref)	
High school	0.850 (0.485, 1.489)	.570
Community college/trade	1.101 (0.651, 1.862)	.719
Some University	1.631 (0.849, 3.132)	.142
Bachelor's degree	2.756 (1.063, 7.145)	.037*
Diagnosis^a^		
CAD	1.00 (ref)	
PCI	0.459 (0.230, 0.915)	.027*
Surgery	0.761 (0.434, 1.333)	.339
HF	0.763 (0.365, 1.596)	.473
MI	1.135 (0.680, 1.893)	.628
Other	0.896 (0.214, 3.755)	.881
Employment Status		
Retired	1.00 (ref)	
Full-time	0.646 (0.359, 1.163	.145
Part-time	1.283 (0.703, 2.338)	.417
Unemployed	1.740 (0.540, 5.611)	.354
Sick leave	2.238 (0.558, 8.982)	.256
Disability	0.813 (0.377, 1.751)	.597
Marital Status		
Married	1.00 (ref)	
Divorced	0.651 (0.338, 1.254)	.199
Widowed	1.804 (0.699, 4.653)	.223
Single	0.682 (0.337, 1.381)	.288

^a^ CAD, coronary artery disease; HF, heart failure; MI, myocardial infarction; PCI, percutaneous coronary intervention. Data are presented as odds ratio (95% CI). The logistic regression model adjusted for all variables listed in the table.

* Alpha set at .05.

**Figure 2:**
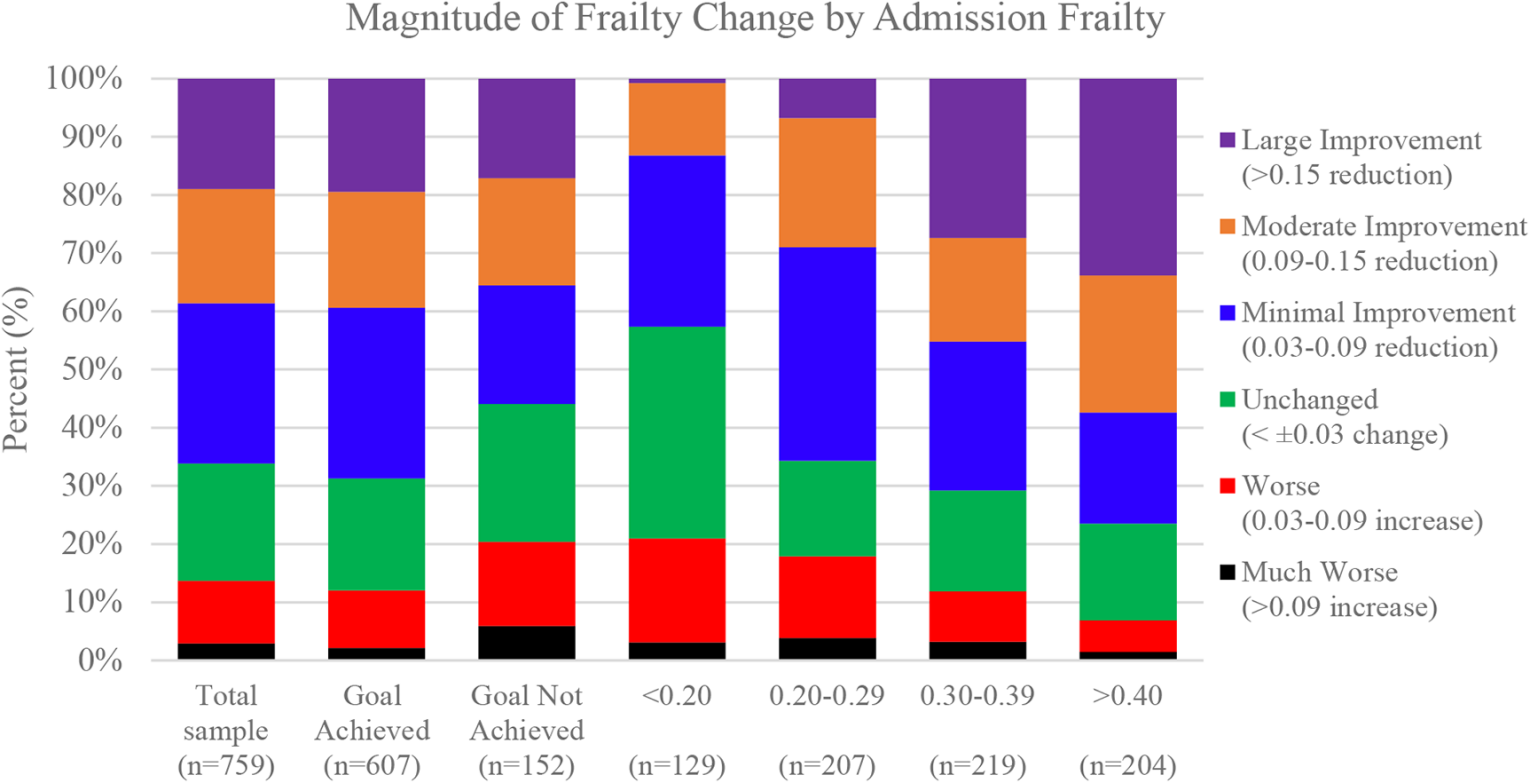
Magnitude of frailty change by admission FI score.

The proportions of FI change in our sample ranged from a 0.22 increase to a 0.39 reduction [mean change: 0.07 (SD = 0.09)]. Among all participants, 290 (27.5%) demonstrated minimally clinically meaningful improvements in frailty, while 149 (19.6%) and 144 (19.0%) experienced moderate (FI reduction of 0.09 to 0.14) and large improvements (≥0.15 improvement), respectively. A total of 153 (20.2%) maintained their initial frailty level (FI ±0.03), while 82 (10.8%) and 22 (2.9%) had worse (0.03–0.09 increase) and much worse frailty scores (>0.09 increase), respectively (Figure 2). Compared to participants who did not achieve a CR goal, a greater proportion of goal achievers had at least a small meaningful improvement in frailty (i.e., FI reduction of 0.03; Figure 2) (19).

## 4 Discussion

The purpose of this study was to examine the influence of frailty on achieving shared goals in CR. Here, we demonstrate a significant inverse association between frailty severity at CR admission and likelihood of CR goal achievement (Table 2). Participants who improved their frailty level by at least 3% (i.e., small but clinically meaningful difference) (19, 20) over the course of their CR program were more than twice as likely to achieve their CR goals compared to those who had worsening frailty (Table 2). Together, we demonstrate that admission frailty and changes in frailty is associated with the attainment of shared goals in CR.

We demonstrate that individuals who did not achieve their CR goal were significantly frailer upon admission to the program (Table 2). For example, we observed that CR participants with lower admission frailty were almost 5 times more likely to achieve their CR goals than those with higher admission FI scores. In fact, for every 1% increase (i.e., one FI unit increase) in admission FI, there was a 3.5% reduced likelihood of achieving a CR goal successfully. Our data suggests that reducing frailty is imperative, not only to improve their health outcomes but to increase the likelihood of achieving their goals. This remains relevant, due to the high proportion of frail individuals enrolling in CR (13, 15, 16, 21–27). In fact, the combined prevalence of frail and severely frail participants accounted for over half (55.8%) of our sample, a proportion that is notably higher than community dwelling older adults (28, 29), but not uncommon for patients with CVD (13, 15, 16, 21–27). Therefore, we suggest admission frailty is an important measure of potential program success and goal-setting, which aligns with our previous work that improving frailty is associated with better health outcomes (13, 16, 23).

Additional research demonstrated that goal-setting was effective among older adults experiencing mild frailty, particularly when a clear need was identified, and realistic goals were linked to functional independence (8). Adhering to these goal setting parameters lead to a positive sense of achievement and concurrently promoted a higher level of independence (8). We relate these findings to our results, which demonstrate that patients who improved their frailty level in CR were more than twice as likely to achieve their goals at the end of the program. It is difficult to determine from our study whether improving frailty was associated with meeting one's goal, or if goal attainment was associated with a reduction in frailty. However, a positive sense of achievement could have synergistic effects to engaging in other health-promoting behaviors. A 3-month quasi-experimental study of an exercise program for frail community-dwelling older adults, which implemented shared goal-setting between participants and their occupational therapist, demonstrated that frailty was reduced at the end of the intervention (30). The authors conclude that life goal-setting techniques are a feasible way to reduce health risk and care needs and frailty levels (30). Overall, we believe our data aligns with the existing literature, and adds new evidence to suggest that understanding patient's frailty changes alongside goal attainment is important to promote healthy cardiovascular behaviors while reducing their risk for adverse health outcomes (16). Thus, with proper intervention and evaluation procedure, improving frailty should become a cornerstone of CR interventions.

### 4.1 Implications and limitations

Frailty has an important role in measuring the admission health and changes in health of CVD patients, and by extension, could become a robust predictor of CR success if frailty measurement is adopted by CR programs. Overall, lower frailty levels, whether observed at CR admission or achieved through improvements in CR, were associated with a higher likelihood of goal achievement, indicating that addressing frailty is important in CR. These findings have implications for CR risk stratification and implementation, emphasizing the need for tailored interventions targeting frailer individuals who stand to benefit the most. Identifying which CR participants may require additional support in CR should help to increase success rates in achieving CR goals.

Nevertheless, our study has limitations to acknowledge. The age of our data and retrospective study design did not allow us to include cognitive contributions to frailty or mitigate the risk of potential biases influencing results, as we were unable to control mechanisms of the CR intervention and data collection, which may have influenced our analysis. Our sample was majority male (76.4%), of working age, and employed full-time (43.9%); data on race was not captured. Our sample predominantly consisted of frail individuals who attended CR, potentially limiting the generalizability of the findings to the general CVD population, some of which would choose not to enrol in CR upon specialist referral. Further, our FI met 4/5 of the criteria described by Searl et al. (31), but its focus on cardiovascular and body composition variables (*n* = 11), along with including only 25 variables instead of the recommended 30–40, may limit its comprehensiveness and precision. The database under study did not collect data on interim clinical events such as hospitalizations or injuries during the CR program, thus limiting key clinical status indicators. Moreover, we acknowledge the absence of data collected on CR participants' adherence to home-based exercise prescription, perspectives of goal attainment, and limited retrospective data on goal evaluation. Finally, our study does not allow us to determine if there is a bi-directional relationship between meeting CR goals and frailty changes. However, our findings suggest that improving frailty is important with respect to goal-attainment. Future research should expand upon these findings by examining how frailty impacts specific CR outcomes as they relate to participants' health, incorporate larger samples from several CR programs (as opposed to one), and prospectively capture data to reduce bias in data collection and interpretation. Furthermore, we recommend future research to consider the social and cultural nuances that may influence frailty levels at CR admission and completion, and determine if such differences have an impact the achievement of outcomes in CR.

## 5 Conclusion

This study provides evidence of the impact of frailty on the achievement of personal health goals in CR. Lower admission frailty, or reducing frailty during CR, significantly improves the likelihood of CR goal attainment, highlighting the importance of frailty assessment in CR programs. Integrating strategies to reduce frailty levels in CR could enhance CR outcomes and contribute to more effective management of CVD in this population.

## Data Availability

The data analyzed in this study is subject to the following licenses/restrictions: “Datasets are available on request. The raw data supporting the conclusions of this article will be made available by the authors, without undue reservation”. Requests to access these datasets should be directed to ev791854@dal.ca.
