# Supplementary material for: The association of frailty on cardiac rehabilitation goal achievement
Source: Front Cardiovasc Med. 2024 Aug 13;11:1441336. doi: 10.3389/fcvm.2024.1441336 (PMC11347271; doi:10.3389/fcvm.2024.1441336)
Supplement: Supplementary file 1 [file Datasheet1.pdf]

Supplementary Table S1. Core components of cardiac rehabilitation[a]

Core Component	Facilitated by	Volume	Material Covered
**Patient Assessment**	Multi-disciplinary led*	Assessments may occur at varying time points (i.e., baseline, completion, throughout), or in a combination of ways.	Graded exercise stress test (i.e., METs), blood lipids, blood sugars, quality of life measures
**Exercise Training**	Physiotherapist, Exercise physiologist	1-3 times weekly, 10-90 minutes in duration	Exercise safety, aerobic and resistance exercises, warm-up and cool-down, monitoring heart rate and rate of perceived exertion
**Goal Setting**	Multi-disciplinary led*	Individual time frames and achievement dates for patients' goals are subject to variability	Examples include weight loss, fitness, cardiovascular health, quality of life, among others
**Nutrition Education**	Dietitian	Weekly, bi-weekly, up to 60 minutes in duration	Healthy food options, controlling blood sugars and lipids, alcohol intake, smoking behaviour
**Medication Education**	Nurse, Family Doctor	Weekly, bi-weekly, up to 60 minutes in duration	Understanding medications. Medical Director provided recommendations on medication changes when necessary.
**Risk Factor Education**	Multi-disciplinary led*	Weekly, 10 minutes-3 hours in duration	Cardiovascular risk factors, smoking behaviours, sedentary behaviour

**Psycho-Social Support**	Multi-disciplinary led*	Weekly, duration based on subjective needs	Guidance on program progression, question and answer opportunities, expectations of the CR program

[a]Table characteristics drawn from North American Guidelines on CR (2).

*Lead by nurse, physiotherapist, dietitian, cardiologist, referring physician, supporting staff (e.g., program lead).

Supplementary Table S2. Frailty index variables used at cardiac rehabilitation admission and completion.

Variable	Cut-off values for deficit
Non-cardiovascular-disease related variables	
Obesity (BMI; kg/m^2)	0= 18.5-25.0<br>0.5= 25.1-29.9<br>1= 30
Waist circumference	Women:<br>0: <=88 cm<br>1: >88 cm<br><br>Men:<br>0: <=102 cm<br>1: >102 cm
SF-36 Physical Function component score<br>SF-36 Role-physical component score<br>SF-36 Bodily Pain component score<br>SF-36 General Health component score<br>SF-36 Energy component score<br>SF-36 Role-emotional component score<br>SF-36 Mental Health component score	 0: >80<br>0.25: 60-80<br>0.5: 40-59<br>0.75: 20-39<br>1: <20
Change in health in the past year	0: Much better; somewhat better; same<br>0.5: Somewhat worse<br>1: Much worse
Percent body fat	Females:<br>0: <35.6%<br>0.33: 35.6-40.9%<br>0.66: 41.0-45.4%<br>1: >45.4%<br><br>Males:<br>0: <24.3%<br>0.33: 24.3-28.6%<br>0.66: 28.7-33.4%

	1: >33.4%
Percent lean muscle mass	Females:<br>0: >17.3%<br>0.33: 15.9-17.3%%<br>0.66: 14.6-17.2%<br>1: >14.6%<br><br>Males:<br>0: >20.4%<br>0.33: 19.1 -20.4%<br>0.66: 17.9 -19.0%<br>1: <17.9%
Food Frequency Score	0: >77<br>0.33: 73-77<br>0.66: 68.5-73<br>1: <68.5
Cardiovascular -disease related variables	
Systolic blood pressure	0: 90-140 mmHg<br>1: <90 or >140 mmHg
Diastolic blood pressure	0: 60-90 mmHg<br>1: <60 or >90 mmHg
Resting heart rate	0: 60-99 bpm<br>1: <60 or >99 bpm
Mean arterial pressure	0: 70-110 mmHg<br>1: <70 or >110 mmHg
Pulse pressure	0: 30-60 mmHg<br>1: <30 or >60 mmHg
Total cholesterol	0: <=6.2 mmol/L<br>1: >6.2 mmol/L
HDL cholesterol	0: >= 1.03 mmol/L<br>1: <1.03 mmol/L
LDL cholesterol	0: 0.98-3.36 mmol/L<br>1: <0.98 or >3.36 mmol/L

Triglycerides	0: <1.67 mmol/L<br>1: >=1.67 mmol/L
Fasting blood glucose	0: 3.9-6.1 mmol/L<br>1: <3.9 or >6.1 mmol/L
NYHA functional class	0: No shortness of breath<br>0.33: Some shortness of breath<br>0.66: Moderate shortness of breath<br>1: Major shortness of breath
Peak metabolic equivalents (METs)	0: >=5 METs<br>1: <5 METs

Abbreviations: BMI, body mass index; bpm, beats per minute; kg, kilograms; m, meters; METs, metabolic equivalents; mmHg, millimetre of mercury; mmol, millimole; SF-36, Short Form Health Survey (36-item).

Supplementary Table S3. Frequency of CR goals by different sample grouping.

Sample Group	Control or lose weight	Physical activity behaviour and fitness	Improve CV risk
**Goal Outcome**			
Achieved CR Goal	251 (41.4%)	216 (35.6%)	140 (23.1%)
Did Not Achieve CR Goal	130 (85.5%)	12 (7.9%)	10 (6.6%)
**FI Admission Groups**			
FI ≤0.19	35 (27.1%)	60 (46.5%)	34 (26.4%)
FI = 0.20-0.29	80 (38.6%)	86 (41.5%)	41 (19.8%)
FI = 0.30-0.39	124 (56.6%)	49 (22.4%)	46 (21.0%)
FI ≥0.40	142 (69.6%)	33 (16.2%)	29 (14.2%)
**Frailty Improvement Groups**			
Much Worse (>0.09 increase)	11 (50.0%)	8 (36.4%)	3 (13.6%)

Worse<br>(0.03-0.09 increase)	47 (57.3%)	22 (26.8%)	13 (15.9%)
Unchanged<br>(< ±0.03 change)	76 (49.7%)	46 (30.1%)	31 (20.3%)
Minimal Improvement<br>(0.03-0.09 reduction)	92 (44.0%)	68 (32.5%)	49 (23.4%)
Moderate Improvement<br>(0.09-0.15 reduction)	77 (51.7%)	48 (32.2%)	24 (16.1%)
Large Improvement<br>(>0.15 reduction)	78 (54.2%)	36 (25.0%)	30 (20.8%)

Data are presented as N, %.

Supplementary Table S4. Demographic information on CR non-completers.

Demographics	Study sample	Total CR non-completers	Admission frailty levels of CR non-completers N=441			
			<0.20	0.20-0.29	0.30-0.39	>0.40
Total (N,%)	*759 (100.0%)*	*502 (100.0%)*	*47 (10.6%)*	*79 (17.9%)*	*106 (24.0%)*	*209 (47.4%)*
Sex						
- Male	580 (76.4%)	363 (72.3%)	37 (78.7%)	64 (81.0%)	75 (70.8%)	144 (68.9%)
Female	179 (23.6%)	139 (27.7%)	10 (21.3%)	15 (19.0%)	31 (29.2%)	65 (31.1%)
Mean age[a] (N, SD)	60.96 (10.84)	60.04 (11.34)	56.3 (10.57)	61.9 (11.38)	60.9 (11.42)	59.1 (11.02)
Frailty Score		N=441				
- <0.2	129 (17.0%)	47 (10.6%)	-	-	-	-
- 0.20-0.29	207 (27.3%)	79 (17.9%)	-	-	-	-
- 0.30-0.39	219 (28.9%)	106 (24.0%)	-	-	-	-
- >0.40	204 (26.9%)	209 (47.3%)	-	-	-	-
Mean Baseline Frailty Score (N, SD)						
Total	*0.32 (0.12)*	*0.39 (0.15)**	0.13 (0.04)	0.25 (0.03)	0.34 (0.03)	0.51 (0.09)
- <0.2	0.14 (0.04)	0.13 (0.04)	-	-	-	-
- 0.20-0.29	0.25 (0.02)	0.25 (0.03)	-	-	-	-
- 0.30-0.39	0.34 (0.03)	0.34 (0.03)	-	-	-	-
- >0.40	0.48 (0.06)	0.51 (0.09)	-	-	-	-
Cardiac Rehabilitation Goal						
- Control or lose weight	381 (50.2%)	172 (34.3%)	11 (23.4%)	17 (21.5%)	41 (38.7%)	80 (38.3%)
- Physical activity behaviour and fitness	228 (30.0%	86 (17.1%)	15 (31.9%)	12 (15.2%)	21 (19.8%)	25 (12.0%)
- Improve CV profile	150 (19.8%)	65 (12.9%)	5 (10.6%)	16 (20.3%)	10 (9.4%)	26 (12.4%)
- Not recorded	0 (0.0%)	179 (35.7%)	16 (34.0%)	34 (43.0%)	34 (32.1%)	78 (37.3%)

Education					34 (	
- No high school	151 (19.9%)	142 (28.3%)	9 (19.1%)	21 (26.6%)	29 (27.4%)	69 (33.0%)
- High school	167 (22.0%)	85 (16.9%)	9 (19.1%)	14 (17.7%)	20 (18.9%)	31 (14.8%)
- Community college/trade	237 (31.2%)	143 (28.5%)	15 (31.9%)	14 (17.7%)	29 (27.4%)	72 (34.4%)
- Some University	130 (17.1%)	89 (17.7%)	8 (17.0%)	20 (25.3%)	17 (16.0%)	23 (11.0%)
- Bachelor’s degree	74 (9.7%)	43 (8.6%)	6 (12.8%)	10 (12.7%)	11 (10.4%)	14 (6.7%)
- Postgraduate	0 (0.0%)	0 (0.0%)	0 (0.0%)	0 (0.0%)	0 (0.0%)	0 (0.0%)
Diagnosis[a]						
- CAD	232 (30.6%)	169 (33.7%)	13 (27.7%)	28 (35.4%)	27 (25.5%)	86 (41.1%)
- PCI	59 (7.8%)	46 (9.2%)	5 (10.6%)	7 (8.9%)	11 (10.4%)	18 (8.6%)
- Surgery	146 (19.2%)	67 (13.3%)	6 (12.8%)	11 (13.9%)	23 (21.7%)	14 (6.7%)
- HF	57 (7.5%)	47 (9.4%)	2 (4.3%)	12 (15.2%)	8 (7.5%)	17 (8.1%)
- MI	252 (33.2%)	158 (31.5%)	19 (40.4%)	18 (22.8%)	33 (31.1%)	72 (34.4%)
- Other	13 (1.7%)	15 (3.0%)	2 (4.3%)	3 (3.8%)	4 (3.8%)	2 (1.0%)
Employment Status						
- Disability	47 (6.2%)	59 (11.6%)	1 (2.1%)	4 (5.1%)	13 (12.3%)	32 (15.3%)
- Sick leave	17 (2.2%)	29 (5.8%)	4 (8.5%)	2 (2.5%)	7 (6.6%)	13 (6.2%)
- Unemployed	34 (4.5%)	25 (5.0%)	4 (8.5%)	5 (6.3%)	4 (3.8%)	8 (3.8%)
- Part-time	155 (20.4%)	124 (24.7%)	16 (34.0%)	24 (30.4%)	21 (19.8%)	50 (23.9%)
- Full-time	333 (43.9%)	179 (35.7%)	11 (23.4%)	36 (45.6%)	38 (35.8%)	67 (32.1%)
- Retired	173 (22.8%)	87 (17.3%)	11 (23.4%)	8 (10.1%)	23 (21.7%)	39 (18.7%)
Marital Status						
- Divorced	56 (7.4%)	62 (12.4%)	6 (12.8%)	7 (8.9%)	8 (7.5%)	37 (17.7%)
- Widowed	52 (6.9%)	35 (7.0%)	2 (4.3%)	7 (8.9%)	8 (7.5%)	13 (6.2%)
- Single	49 (6.5%)	45 (9.0%)	4 (8.5%)	8 (10.1%)	9 (8.5%)	21 (10.0%)
- Married	602 (79.3%)	360 (71.7%)	35 (74.5%)	57 (72.2%)	81 (76.4%)	138 (66.0%)

[a]Abbreviations: CAD, coronary artery disease; CV, cardiovascular; HF, heart failure; MI, myocardial infarction; N, number; PCI, percutaneous coronary intervention; QOL, quality of life; SD, standard deviation.

Supplementary Table S5. Magnitude of Frailty Change by Admission FI

Frailty Change	Total sample	Goal Achieved	Goal Not Achieved	<0.20	0.20-0.29	0.30-0.39	>0.40
Much Worse (>0.09 increase)	22	13	9	4	8	7	3
Worse (0.03-0.09 increase)	82	60	22	23	29	19	11
Unchanged (< ±0.03 change)	153	117	36	47	34	38	34
Minimal Improvement (0.03-0.09 reduction)	209	178	31	38	76	56	39
Moderate Improvement (0.09-0.15 reduction)	149	121	28	16	46	39	48
Large Improvement (>0.15 reduction)	144	118	26	1	14	60	69
**Total**	**759**	**607**	**152**	**129**	**207**	**219**	**204**

Supplementary Table S6. Demographics of participants according to frailty change.

Demographics	Study Sample	Frailty improvement groups					
		Much Worse (>0.09 increase)	Worse (0.03-0.09 increase)	Unchanged (<±0.03 change)	Minimal (0.03-0.09 reduction)	Moderate (0.09-0.15 reduction)	Large (>0.15 reduction)
Total (N,%)	*759 (100.0%)*	*22 (2.8%)*	*82 (10.8%)*	*153 (20.1%)*	*209 (27.5%)*	*149 (19.6%)*	*144 (18.9%)*
Sex							
- Male	580 (76.4%)	18 (81.8%)	56 (68.3%)	121 (79.1%)	161 (77.0%)	114 (76.5%)	110 (76.4%)
Female	179 (23.6%)	4 (18.2%)	26 (31.7%)	32 (20.9%)	48 (23%)	35 (23.5%)	34 (23.6%)
Mean age[a] (N, SD)	60.96 (10.84)	61.23 (8.24)	62.3 (11.77)	61.8 (10.73)	60.2 (11.24)	60.8 (10.42)	60.6 (10.62)
Frailty Score							
- <0.2	129 (17.0%)	-	-	-	-	-	-
- 0.20-0.29	207 (27.3%)	-	-	-	-	-	-
- 0.30-0.39	219 (28.9%)	-	-	-	-	-	-
- >0.40	204 (26.9%)	-	-	-	-	-	-
Mean Baseline Frailty Score (N, SD)							
Total	*0.32 (0.12)*	0.28 (0.10)	0.27 (0.13)	0.29 (0.14)	0.30 (0.11)	0.34 (0.12)	0.41 (0.10)
- <0.2	0.14 (0.04)	-	-	-	-	-	-
- 0.20-0.29	0.25 (0.02)	-	-	-	-	-	-
- 0.30-0.39	0.34 (0.03)	-	-	-	-	-	-
- >0.40	0.48 (0.06)	-	-	-	-	-	-
Cardiac Rehabilitation Goal							
- Control or lose weight	381 (50.2%)	11 (50.0%)	47 (57.3%)	76 (49.7%)	92 (44.0%)	77 (51.7%)	78 (45.2%)
- Physical activity behaviour and fitness	228 (30.0%	8 (36.4%)	22 (26.8%)	46 (30.8%)	68 (32.5%)	48 (32.2%)	36 (25.0%)
- Improve CV profile	150 (19.8%)	3 (13.6%)	13 (15.9%)	31 (20.3%)	49 (23.4%)	24 (16.1%)	30 (20.8%)
Education							
- No high school	151 (19.9%)	3 (13.6%)	19 (23.2%)	39 (25.5%)	39 (18.7%)	27 (18.1%)	24 (16.7%)
- High school	167 (22.0%)	8 (36.4%)	23 (28.0%)	27 (17.6%)	45 (21.5%)	32 (21.5%)	32 (22.2%)
- Community college/trade	237 (31.2%)	7 (31.8%)	24 (29.3%)	47 (30.7%)	63 (30.1%)	52 (34.9%)	44 (30.6%)
- Some University	130 (17.1%)	2 (9.1%)	10 (12.2%)	23 (15.0%)	44 (21.1%)	26 (17.4%)	25 (17.4%)
- Bachelor's degree	74 (9.7%)	2 (9.1%)	6 (7.3%)	17 (11.1%)	18 (8.6%)	12 (8.1%)	19 (13.2%)
- Postgraduate	0 (0.0%)	0 (0.0%)	0 (0.0%)	0 (0.0%)	0 (0.0%)	0 (0.0%)	0 (0.0%)

Diagnosis[a]							
- CAD	232 (30.6%)	3 (13.6%)	23 (28.0%)	53 (34.6%)	67 (32.1%)	40 (26.8%)	46 (31.9%)
- PCI	59 (7.8%)	3 (13.6%)	3 (3.7%)	17 (11.1%)	15 (7.2%)	11 (7.4%)	10 (6.9%)
- Surgery	146 (19.2%)	3 (13.6%)	16 (19.5%)	27 (17.6%)	45 (21.5%)	29 (19.5%)	26 (18.1%)
- HF	57 (7.5%)	3 (13.6%)	10 (12.2%)	11 (7.2%)	12 (5.7%)	11 (7.4%)	10 (6.9%)
- MI	252 (33.2%)	8 (36.4%)	28 (34.1%)	41 (26.8%)	68 (32.5%)	56 (37.6%)	51 (35.4%)
- Other	13 (1.7%)	2 (9.1%)	2 (2.4%)	4 (2.6%)	2 (1.0%)	2 (1.3%)	1 (0.7%)
Employment Status							
- Disability	47 (6.2%)	1 (4.5%)	7 (8.5%)	14 (9.2%)	11 (5.3%)	8 (5.4%)	6 (4.2%)
- Sick leave	17 (2.2%)	3 (13.6%)	2 (2.4%)	3 (2.0%)	3 (1.4%)	2 (1.3%)	4 (2.8%)
- Unemployed	34 (4.5%)	1 (4.5%)	3 (3.7%)	10 (6.5%)	6 (2.9%)	7 (4.7%)	7 (4.9%)
- Part-time	155 (20.4%)	4 (18.2%)	19 (23.2%)	23 (15.0%)	49 (23.4%)	27 (18.1%)	33 (22.9%)
- Full-time	333 (43.9%)	9 (40.9%)	35 (42.7%)	67 (43.8%)	98 (46.9%)	68 (45.6%)	56 (38.9%)
- Retired	173 (22.8%)	4 (18.2%)	16 (19.5%)	36 (23.5%)	42 (20.1%)	37 (24.8%)	38 (26.4%)
Marital Status							
- Divorced	56 (7.4%)	3 (13.6%)	8 (9.8%)	14 (9.2%)	13 (6.2%)	8 (5.4%)	10 (6.9%)
- Widowed	52 (6.9%)	1 (4.5%)	7 (8.5%)	9 (5.9%)	17 (8.1%)	9 (6.0%)	9 (6.3%)
- Single	49 (6.5%)	1 (4.5%)	6 (7.3%)	12 (7.8%)	10 (4.8%)	10 (6.7%)	10 (6.9%)
- Married	602 (79.3%)	17 (77.3%)	61 (74.4%)	118 (77.1%)	169 (80.9%)	122 (81.9%)	115 (79.9%)

[a]Abbreviations: CAD, coronary artery disease; CV, cardiovascular; HF, heart failure; MI, myocardial infarction; N, number; PCI, percutaneous coronary intervention; QOL, quality of life; SD, standard deviation.

Supplementary Table S7. Sensitivity analysis – odds of CR goal achievement by magnitude of frailty change.

Variable	Adjusted Odds Ratio (OR, 95% CI)	P-value
Frailty Improvement Group		
- ≥0.03 frailty increase	1.00 (ref)	(ref)
- ±0.03 frailty change	1.408 (0.772, 2.569)	.264
- ≥0.03 frailty reduction	2.111 (1.262, 3.532)	*.004**
Frailty improvement per 0.01-unit change	1.027 (1.005, 1.048)	*.014**

SupplementaryFigures:

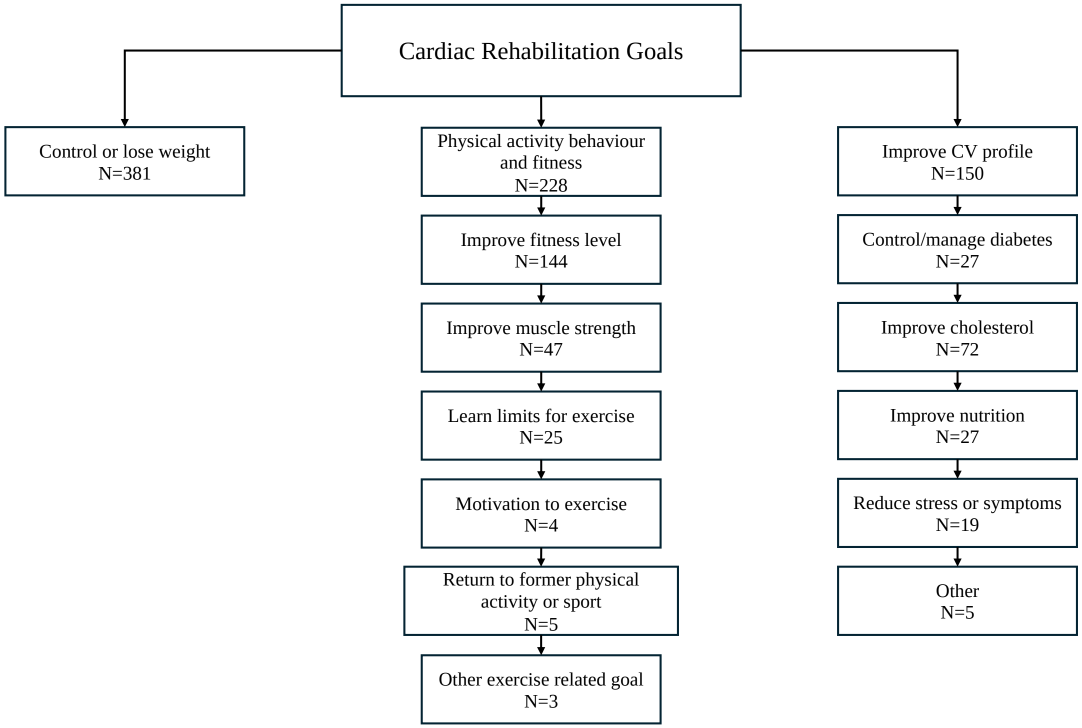

Supplementary Figure S1. Flowchart of how cardiac rehabilitation goals were collapsed into distinct groups.
